# The Use of Terrestrial Laser Scanning for Determining the Driver’s Field of Vision

**DOI:** 10.3390/s17092098

**Published:** 2017-09-13

**Authors:** Tomáš Zemánek, Miloš Cibulka, Petr Pelikán, Jaromír Skoupil

**Affiliations:** 1 Department of Forest and Forest Products Technology, Faculty of Forestry and Wood Technology, Mendel University in Brno, Brno 61300, Czech Republic; 2 Department of Forest Management and Applied Geoinformatics, Faculty of Forestry and Wood Technology, Mendel University in Brno, Brno 61300, Czech Republic; milos.cibulka@mendelu.cz; 3 Department of Landscape Management, Faculty of Forestry and Wood Technology, Mendel University in Brno, Brno 61300, Czech Republic; petr.pelikan@mendelu.cz; 4 Department of Horticultural Technology, Faculty of Horticulture, Mendel University in Brno, Brno 61300, Czech Republic; jaromir.skoupil@mendelu.cz

**Keywords:** terrestrial laser scanner, driver’s field of vision, tractor, harvester

## Abstract

Terrestrial laser scanning (TLS) is currently one of the most progressively developed methods in obtaining information about objects and phenomena. This paper assesses the TLS possibilities in determining the driver’s field of vision in operating agricultural and forest machines with movable and immovable components in comparison to the method of using two light point sources for the creation of shade images according to ISO (International Organization for Standardization) 5721-1. Using the TLS method represents a minimum time saving of 55% or more, according to the project complexity. The values of shading ascertained by using the shadow cast method by the point light sources are generally overestimated and more distorted for small cabin structural components. The disadvantage of the TLS method is the scanner’s sensitivity to a soiled or scratched cabin windscreen and to the glass transparency impaired by heavy tinting.

## 1. Introduction

Drivers of agricultural and forest machinery and/or equipment for earth-moving work operate movable machine components such as front-end adapters for handling the load with attached or mounted devices—often in the environment with natural or artificial obstacles requiring good orientation. In such conditions, the quality of the vision from the cabin of these operating machines affects not only the drivers’ work performance but also the safety of their labour. A driver’s poor vision of the operating machine caused by an inappropriate design of the driver’s cabin of a movable component can lead to property damage or industrial accidents. A survey conducted by Caisse Nationale d’Assurance Maladie (French National Insurance Fund) on earth-moving machines showed, for example, that a third of serious and fatal accidents are caused by the lack of visibility from the driver’s cab [1].

Pursuant to the interpretation of the ISO 5721-1 standard, the field of vision is an “area, which can be seen by the eye from the position of a sitting operator”. We can distinguish a direct field of vision (given by direct visibility) and an indirect field of vision (assisted by mirrors or other visual aids). The determination of the driver’s field of vision is one of the acts performed according to the valid legislation on approving the type of selected vehicle categories. The list below includes a selection of legislative measures related to the driver’s field of vision valid in the European Union.

As to the field of vision and the front screen wiper, the wheeled and caterpillar tractors must meet the requirements of the standards of ISO 5721-1:2013 Agricultural tractors–Requirements, test procedures and acceptance criteria for the operator’s field of vision—Part 1: Field of vision to the front [2], and ISO 5721-2:2014 Agricultural tractors–Requirements, test procedures and acceptance criteria for the operator’s field of vision—Part 2: Field of vision to the side and to the rear [3]. The quoted standards conform to the Commission delegated Regulation (EU) 2015/208 of 8 December 2014 [4] supplementing Regulation (EU) No. 167/2013 of the European Parliament and of the Council with regard to vehicle functional safety requirements for the approval of agricultural and forestry vehicles and supervision over the market with these vehicles [5]. Harvester and forwarder operator’s field of vision is also normally determined in line with the above standards. Requirements for the field of operator’s vision in the wheeled and caterpillar tractors are stipulated by the Directive of the European Parliament and of the Council No. 2008/2/EC of 15 January 2008 on the field of vision and windscreen wipers for wheeled agricultural and forestry tractors [6]. Requirements for the field of vision of the operators of earth-moving machines are defined in the standard ISO 5006:2017 Earth-moving machinery—Operator’s field of view—Test method and performance criteria [7]. Vehicles of M1 category (categories specified by Directive of the European Parliament and of the Council No. 2007/46/EC [8]) have the driver’s field of vision to the front determined by a different procedure, viz. by determining the dimensional relations between points in a 3D reference grid according to the Regulation No. 125 of the Economic Commission for Europe of the United Nations (UN/ECE)—Uniform provisions concerning the approval of motor vehicles with regard to the forward field of vision of the motor vehicle driver [9].

The driver’s field of vision is most frequently determined by practical methods frankly on the machine in the terrain. A widespread practical method is to use one or two light point sources placed in a pre-defined position relative to the reference point of the cabin seat. Subsequently, there is a shade image (the deepest shade), which overlays projected onto the semi-circular vision to the front (the chords of the obstructing sphere of shade). If one light source is used, it must be placed gradually into both positions symmetrical in relation to the reference point of the seat. The methodological procedure for agricultural tractors is given in the standard ISO 5721-1 [2]. A vision obstruction can be recorded manually by chalk onto the horizontal surface with a designated square grid; a geodetic apparatus, camera or cine-camera can also be used. For the recording of the practically determined shading, Barron et al. [10] used a network of sensors distributed within the semi-circular vision, which recorded the intensity of the incident of light. Thus, the authors exactly defined the often-subjective determination of the boundary of the deepest shade of a shade image. Hella et al. [1] compared the method using light point sources with the photographic method of determining the semi-circular driver’s vision. From their study, they are in favor of the whole-area analysis of the semi-circular vision because of its greater informative value.

Another practical method uses two lasers with parallel beams placed in a standard defined position relative to the reference point of the seat. The laser beams automatically draw a circle with a radius of 12 m and the laser angle shift from the basic position is recorded when the beams collide with the structural elements of the cabin. The position and length of the shading section chord on the circle line is then determined based on these values. The method is used by the Government Testing Laboratory of Machines [11].

The method of terrestrial laser scanning discussed in this paper is one of the practical procedures to determine also the driver’s field of vision. The TLS principle is based on the optical laws of space penetration by light rays, their impassability through non-transparent obstacles therein and consists of the transmission of a great amount of laser impulses in various directions. These impulses are captured by solid obstructions to the driver’s vision while passing freely through the inside and outside of the cab at places where the driver’s vision is not obstructed. Thus, it is created in the plane of the stationary machine image of the field of vision. The most suitable measurement is the mobile 3D scanner with a swivel base, mounted on a stand in the driver’s cabin into two standard defined positions that are parallel in relation to the reference point of the seat.

A disadvantage of these practical methods for establishing the driver’s field of vision is the fact that they can be applied only after the machine has been manufactured. If a need arises at this moment for essential change of the cabin design, it may be a rather costly problem. This is where the theoretical methods of determining the driver’s field of vision enter the game.

One of the basic theoretical procedures is the mathematic determination of shading for binocular vision. The method is described in detail in standards ISO 5721-1 [2] or ISO 5006 [7].

A frequently used group of theoretical methods is the analysis of the driver’s vision in CAD (Computer-Aided Design) programme systems. The basic prerequisite for such a procedure is the use of project documentation of the machine. These methods are fast and the application of design changes does not represent high costs as in the case of a machine in batch production. The driver’s field of vision can be analysed simply from different user-defined positions corresponding to the performance of various working operations, e.g., driving with a load or turning. Ryan et al. [12] analysed the driver’s field of vision during work operations with a hydraulic crane using a virtual model of human figures of different heights. Choi et al. [13] compared the driver’s field of vision determined by using a digital human body model with the methods of light point source and with individual visibility tests of six helpers. Gilad and Byran [14] evaluated the driver’s field of vision in three tractor models by using the innovated method of a virtual human body model.

A disadvantage of the theoretical procedures for determining the driver’s field of vision is the fact that results sometimes do not correspond to reality due to manufacturing tolerances of machines and the possibilities of additional equipment.

The goal of this study was to assess possibilities of terrestrial laser scanning for determining the driver’s field of vision to the front in accordance with the standard ISO 5721-1 [2].

## 2. Related Work

The practical analysis of the driver’s field of vision based on the creation of a shade image with the use of two light point sources is frequently applied in mobile technology. The method is relatively time demanding as to the data collection and the evaluation as well as in meeting the necessary conditions (the presence of a square grid in the projection surface, etc.). To identify the cast of shade images, the measurement must be done at night or in an enclosed area without day light access. Even so, determining the boundaries of the deepest shade is sometimes problematic. Moreover, moving the operating machines to another place or interrupting their work for the measurements is rather costly. The development of advanced measuring techniques brings new possibilities for how to make the determination of the driver’s field of vision faster, more accurate and easier. The employment of a 3D laser scanner can be such an option.

Terrestrial laser scanning (TLS) is currently one of the most progressive methods to obtain information about objects and phenomena on, above or under the earth’s surface. It is the latest technology of 3D data collection. By using this technology, users obtain a great amount of actual data at a very high resolution and within a very short time. Core areas of the recent use of this method—there are documentations about the actual condition of buildings (Tang et al. [15]; Shukor et al. [16]; Fais et al. [17], natural elements (Carrivick et al. [18]; Griebel et al. [19]; Kitov et al. [20]; Spreafico et al. [21]; Kong et al. [22] or modeling of complex objects such as industrial premises, archaeological sites or underground areas (Torres et al. [23]; Hullo et al. [24]; Gallay et al. [25]).

Mapping the tower-crane operator’s field of vision for improved labour safety at building sites, Cheng and Teizer [26] and Shapira et al. [27] used the method of terrestrial laser scanning while placing the apparatus outside the crane cabin. Bhattacherya et al. [28] employed the TLS method to analyse the mine loader operators’ field of vision. When repeating the measurement on the same machine, their results corresponded to 96%, which was by 3% more than in the test conducted by using the method of light point sourcing. West et al. [29] compared the analysis of the mine loader operator’s field of vision by TLS and CAD methods. TLS values of total visibility obtained within the field of vision to the front were lower by 15.5%. The authors attributed the difference to the inappropriate operational setting of the scanner for the TLS, to its misplacement in the cabin relative to the reference point of the seat or to the manufacturing tolerance of the machine dimensions.

## 3. Materials and Methods

**Figure 1:**
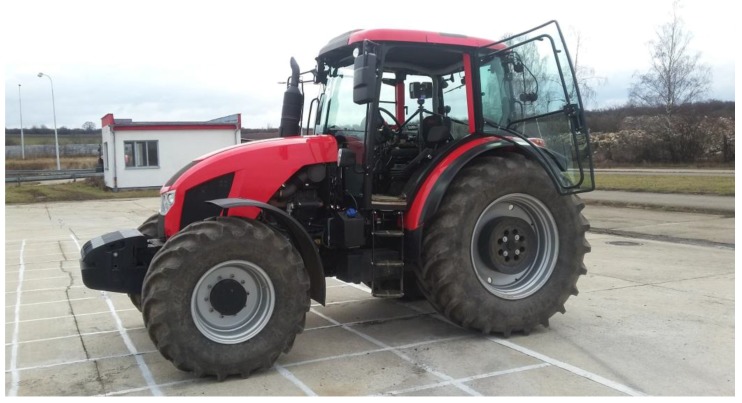
Agricultural tractor Model Zetor Forterra 150 HD, Brno, Czech Republic.

**Figure 2:**
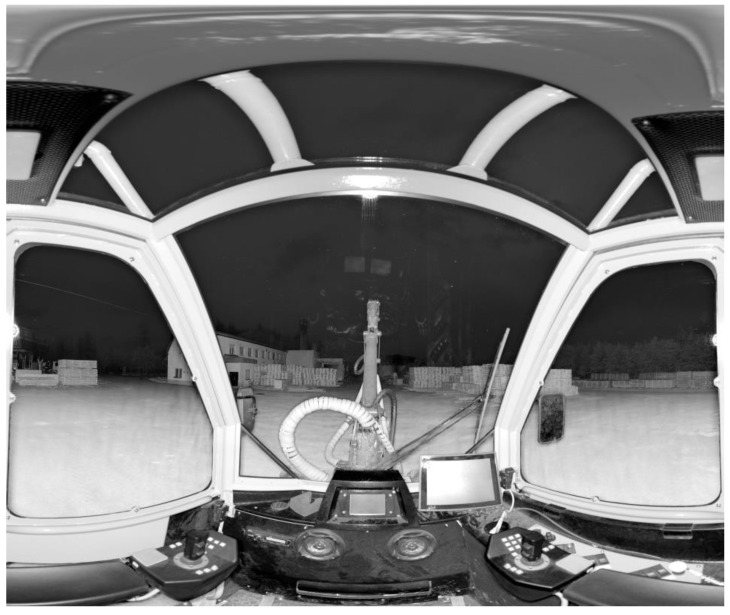
Driver’s field of vision from harvester Model H50 Strojírna Novotný, Vitošov, Czech Republic (photo from scanner).

The method of establishing the driver’s field of vision by terrestrial laser scanning according to ISO 5721-1:2013 was tested on three types of working machines: the agricultural tractor, the harvester and the forwarder. In this study, the TLS method is presented on the agricultural tractor Model Zetor Forterra 150 HD (Figure 1) as on an operating machine with immovable components, and on the harvester Model H50 made by Strojírna Novotný (Figure 2), representing an operating machine with movable tools. In both machines, the results are presented by means of a driver’s forward field of vision. In the third tested machine—a forwarder—the position of the hydraulic crane on the machine’s rear half-frame affects the driver’s backwards field of vision, and this is why we deal only with the agricultural tractor and harvester in the following text.

The agricultural tractor was measured in the presence of Zetor Tractors a.s. representatives in the test polygon of the corporate development testing laboratory in Brno. The company Zetor Tractors a.s. provided a comparison of their own results from the measurements of semi-circular field of the driver’s vision to the front determined by the casting of shade from two point sources according to the same above-mentioned standards. The harvester driver’s field of vision was measured at the technical grounds of the Training Forest Enterprise Masaryk Forest in Křtiny, an organizational unit of Mendel University in Brno. The field of vision was determined by the TLS method and by the method of the two light point sources. The hydraulic crane of the harvester was arranged in a position parallel to the longitudinal machine’s axis with the distance of the harvester head’s vertical axis from the vertical axis of the hydraulic crane pillar being 5 m.

The instrument used for the measurements was the static scanner of the “Phase-shift” type, which determines the position of points based on the continual measurement of the phase shift between the laser beams transmitted and received [30]. The length range of the used Faro Focus 3D scanner is from 0.6 to 120 m and its aiming capacity is up to 976 thousand points per second. In addition to the full resolution (1/1), the scanner also allows work at a lower resolution (1/2, 1/4, 1/5, 1/8, 1/10, 1/16, 1/32). Depending on the resolution, the scanning quality can be adjusted from 1× to 8× (number of measurements taken on each point). For the measurement, the scanner uses radiation with a wavelength of 905 nm. The accuracy of the length determination is ±2 mm per 10 m, and the horizontal and the vertical range of measurements are 360° and 305°, respectively.

**Figure 3:**
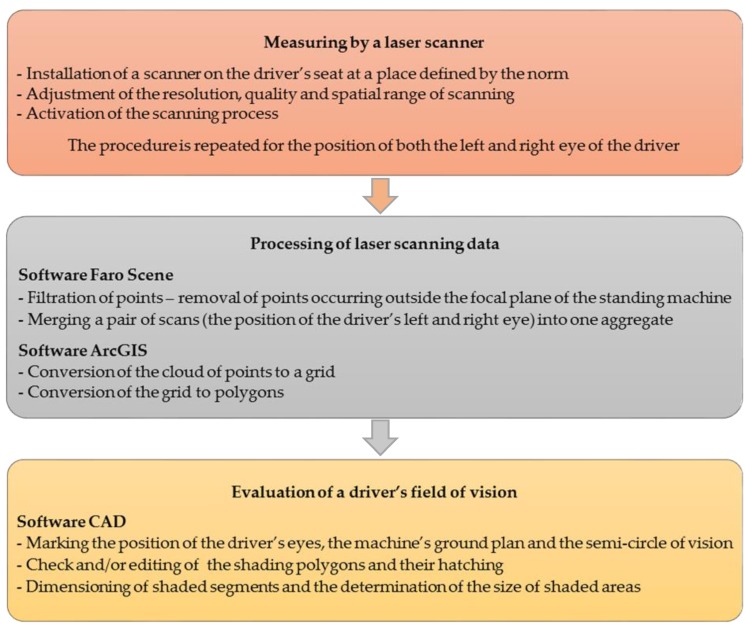
Scheme illustrating the TLS method procedure.

The workflow can be divided into three basic steps. For better clarity, a scheme of the below-described procedure is added in Figure 3.

### 3.1. Measuring by a Laser Scanner

The first step consisted of the placement of four spherical targets (min. number being three) behind the segment of a circle with a radius of 12 m in the test polygon. The targets serve for the post-processing connection of scanning positions from the place of the left and right eye. After placing the center of the scanner head to the standard defined place on the driver’s seat, it was necessary to adjust the resolution, the quality and the spatial range prior to the scanning. One measuring position was used to take three scans at resolution 1/2, 1/4 and 1/8. A lower resolution does not provide a sufficiently accurate display of the smaller details. Distances between the points in the cloud of points are greater (more than 12 mm at a distance of 10 m from the scanner) and the required details do not show at all or only partially. The quality of the scanning for these three resolutions were set to the mean value of 4×, which guaranteed a sufficient quality of measurement at a relatively rapid scanning. The reason for choosing only one (mean) quality value was primarily time intensity of measurements (limited time of possibility to use the working machines) as well as findings from measurements in other projects. In testing the adjustment of different quality values (2, 4 and 6), the noise in the clouds of points was nearly identical and thus did not affect their resulting accuracy. The spatial range of measurement was adjusted from 0° to 180° in the horizontal direction and from –60° to 90° in the vertical direction. The direction of the scanner turning during the scanning was clockwise. This is why zero was set on the left in the horizontal range (from the perspective of the tractor driver), perpendicular to the direction of forward travel. As required by the standard, the scans were taken gradually at the two scanner positions (the distance of axes 65 mm) symmetrical with respect to the reference point of the driver’s seat.

### 3.2. Processing of Laser Scanning Data

After the end of the scanning, the data was imported from the scanner SD card into the Faro Scene basic processing software (version 5.3, Faro Technologies, Inc., Lake Mary, FL, USA) [31] supplied along with the scanner. The first step consisted in the automated filtration based on the "Stray" filter to eliminate so-called stray (faulty) points. Stray filter remove scan points resulting from hitting two objects with the laser spot or hitting no object at all, for example the sky [31]. The next step was the filtration during which all points were manually removed whose heights were greater than that of the points in the imaging horizontal plane (terrain surface) and the points imaging of the machine itself (the agricultural tractor, the harvester). These steps were made for all three resolutions. Now what remained were the points that occurred in the referenced imaging plane covering the area visible from the driver’s place. In the subsequent step, the pairs of scans with the same resolution were merged into one file and the files were exported into the selected format.

**Figure 4:**
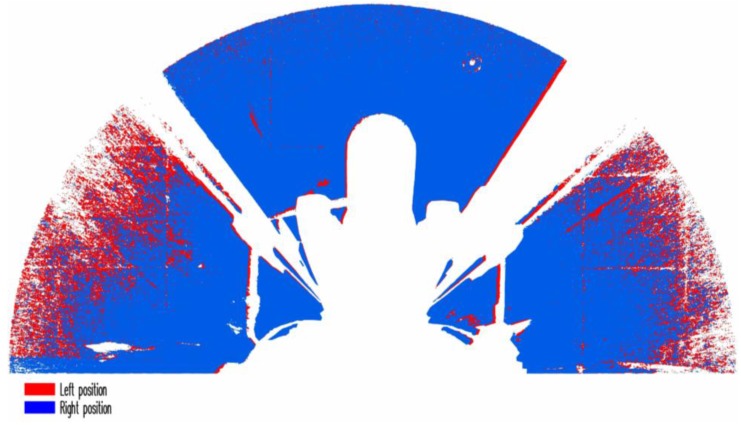
Merged clouds of points from the left and right scanner position converted to polygons (agricultural tractor).

Processing in the ArcGIS programme (version 10.3.1, ESRI, Redlands, CA, USA) was a step between the Faro Scene and the CAD programmes. The import of data into ArcGIS with the highest test resolution (1/2) was impossible due to the file size. Therefore, a new export of points in the Faro Scene programme had to be made with the row and column values changed from 1 to 2 prior to the export (point clouds subsampled with a factor 4). In practice, this means that the exported cloud of points was thinned. Nevertheless, the density of this cloud of points is higher after the thinning than that of the original non-reduced cloud of points obtained at a resolution of 1/4. After this step, the file with the highest resolution could be imported successfully. In the subsequent step, the layer of points was transferred onto the raster by using the “Feature To Raster” (FTR). For the successful implementation of this step, an attribute field had to be added in the point layer table of attributes and a numerical value filled in, e.g., 1. Before running the FTR tool, the created attribute field was entered into the *Field* parameter and the raster cell size was selected. After the implementation of this step, a check was made whether the raster corresponded to our expectations. Primarily, whether the raster was not created also at places, which were invisible from the driver’s seat (obscure sites). If the raster was not correct in terms of imaging, we changed the raster cell size and ran the FTR tool again. In the subsequent step, the raster was transferred into the polygon by using the “Raster To Polygon” tool. This tool converts a raster dataset to polygon features. CAD is a programme primarily for working with vector datasets and this is why the work with the loaded layer of polygon (vector format) is considerably easier and faster than with the grid. A cloud of points created by merging two scans (from the position of the driver’s left and right eye) converted into the polygon vector format is presented in Figure 4.

Output data of the 3D scanning process was exported from the GIS environment in the DXF (Drawing Exchange Format) exchange format, which has both the text ASCII (American Standard Code for Information Interchange) and the binary form and hence is supported by a wide range of industry-specific specialized programmes. Imaging and further processing of the data occurred in CAD systems through a combination of automated and manual processes.

### 3.3. Evaluation of a Driver’s Field of Vision

In order to enhance the labour efficiency in the recurrent data processing, we developed a drawing template (DWT). This template was prepared on individual level, intended for the classification of new drawing objects coming into existence during the analysis, the styles of text labels and the dimension of figures as well as the layout for a quick printing of the result drawing or to export it into a more convenient portable data file. The template further includes vector objects of unchangeable parts of the output on a scale corresponding to the output from the 3D scanner. It is a ground plan view of the machine with the marked location of the driver’s eyes at the beginning of the coordinate system, the sector of the vision to the front as an external extension of the sector of the semi-circular vision with a chord of 9.50 m in length, and a circular sector with a central angle of 180° and radius of 12 m (semi-circular), representing the analysed area vision.

In accordance with the used standard, the vision obstruction was illustrated in the diagram of the semi-circular driver’s vision to the front by marking the chords of the semi-circular vision to the front, which cannot be seen because of the machine’s constructional elements. Subsequently, the chords were dimensioned by using the *Dimaligned* tool.

It was beyond the framework of the requirement standards for a more detailed analysis of the overall shading, the shading by movable machine adapters (hydraulic crane of harvester) and the shading by the front corner posts of the cabin in the area of the semi-circular driver’s vision. For these purposes, we used the *Hatch* tool, the results of which are objects for which the software always computes the area from the definition of their boundaries. The result is information about the total obstruction vision with no regard to the more detailed differential of the individual parts of the machine. In line with the requirement of the standard ISO 5721-1:2013, the area occurring in the ground plan view at a distance greater than 12 m from the centre of the scanner head (the position of the driver’s eyes) had to be filtered off. The calculation of the areas by means of hatching the sections delineated by the semi-circular sector and by the inner boundaries of shading resolved at the same time the problem with the data occurring outside the examined area. This also eliminated the problem with small polygons inside the larger continuous areas of shading, which represented the so-called “holes” in the surface, created during the data processing in GIS due to the adjusted value of the maximum length of triangle sides within the TIN (Triangulated Irregular Network).

In some cases, to determine the area of the cast of shade, we had to add manually the boundaries in the form of curves in shading sectors between the cabin, the movable adapters and the corner posts with grab handles. The thematically related polygons were hatched and consequently their areas were established.

## 4. Results

The accuracy of the scanner used in the terrestrial laser scanning method is two orders higher than required by the standard. The most convenient form for the subsequent work with the data appeared to be the parameter of resolution 1/2 (high) in combination with the parameter of quality 4× (medium), which guarantees a good ratio between the scanning speed and the quality of the data obtained. At the resolution ½, the scanned points would create a network of points with a spacing of 3.68 mm at a distance of 12 m from the scanner. The spacing of points increases proportionally with the increased distance. At the lower resolutions 1/4 and 1/8, the scanning was faster, but the imaged points showed larger spacing (7.36 resp. 14.73 mm) at a distance of 12 m from the scanner. This is why a larger cell size had to be chosen in order to create a continuous raster, by which the raster appeared also at places of non-shaded areas, thus distorting the results of the surface image of the shading.

**Figure 5:**
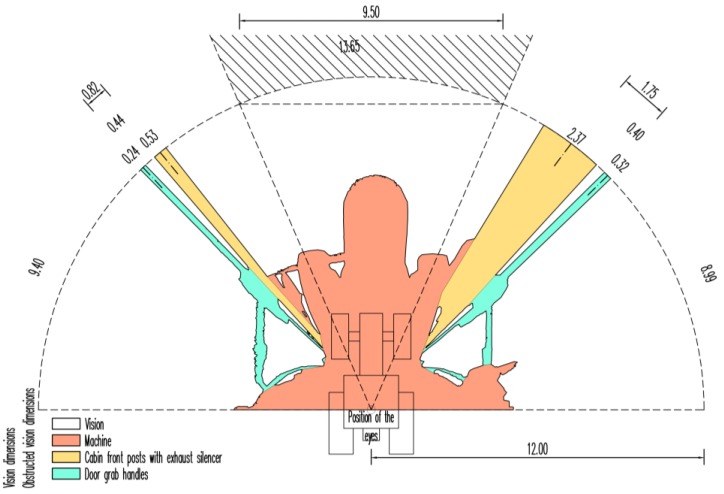
Semi-circular vision to the front of agricultural tractor driver determined by the TLS method. Note: dimensions are in *m*.

**Figure 6:**
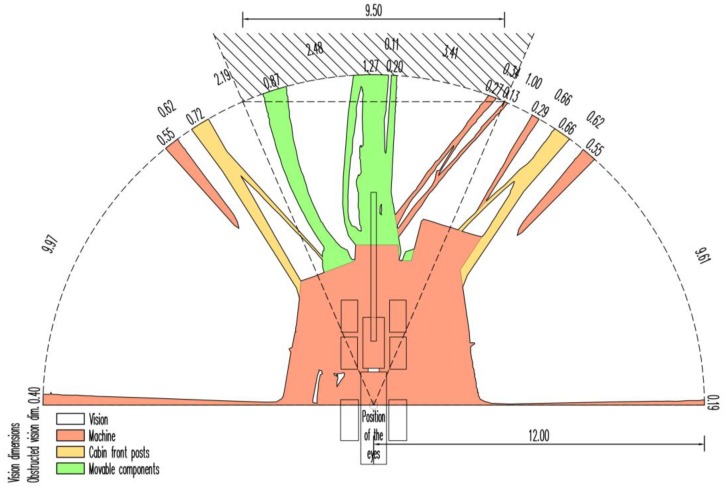
Semi-circular vision to the front of harvester driver determined by the TLS method. Note: dimensions are in *m*.

The output of the analysis in the CAD software is a ground plan view of the agricultural tractor and the harvester with a particular semi-circular sector and a sector of vision to the front created as an external extension of the sector of the semi-circular vision with a chord of 9.50 m in length (Figure 5 and Figure 6). In the diagrams, the chords of shading caused by the machine’s constructional elements and the distances between the centres of the two neighbouring shadings are dimensioned. For clarity, the metre dimensions along the arc segment of the particular area are expressed with an accuracy of cm. The white sectors represent open areas of the driver’s vision, which are projected into the terrain surface. The hatch patterns distinguish the areas of shading caused by the machine’s constructional elements.

The graphical output is further supplemented by results (Table 1 and Table 2) in table form with a legend. The total obscure area in the vision of the agricultural tractor driver within a semi-circular sector with a radius of 12 m was 60.60 m^2^ (26.79%). Of this, 16.02 m^2^ fell to the left and right front posts of the tractor cabin including the exhaust silencer, and 7.10 m^2^ fell to the grab handles of the right and left cabin doors. The total area of the driver’s vision was 165.60 m^2^. The sum of segments (chord lengths) corresponding to the obstruction of the driver’s vision was 3.46 m, which is 9.52% of the total value of the sum of the shaded segments and the driver’s vision on the boundary line of the semi-circular sector with a radius of 12 m. The longest visual obstruction of 2.37 m fell to the right post of the cab, which optically connected with the shading of the exhaust silencer. An essential change in the harvester driver’s field of vision as compared with the agricultural tractor was the presence of the hydraulic crane. The total area of the harvester driver’s vision obstruction at the given position of the crane in the semi-circular sector with a radius of 12 m was 64.86 m^2^ (28.67%). Of this, 13.44 m^2^ went to the hydraulic crane and the hydraulic hoses. The total area of the harvester driver’s vision was 161.34 m^2^.

## 5. Discussion

We have to mention some findings and moments from developing the procedure of the partial data processing in the CAD software, which can influence the resulting values and the quality of the analysis. The principle of data adaptation in the GIS environment dwells on the interpolation of a cloud of points into the irregular triangular network with the subsequent transfer into polygons. Hence, the data did not provide smoothed outer boundaries of polygons. For this reason, it was necessary to work with data reaching beyond the boundary of the 12 m ground plan distance view from the driver’s eyes and to consider the fact already at scanning with a 3D scanner and the data adaptation in GIS. The problem has a simple solution in the CAD environment, which consists of trimming off the data along the semi-circular sector boundary, by which the rounded segments will arise on the boundary of the analysed surface.

Emphasis is placed on the greatest possible automation of processes leading to the determination of the resulting values and the graphic design of the vision and the vision obstruction. However, full automation was not achieved due to the nature of the used software in which the user selects the tools and defines the objects for their creation or modification during the work. In CAD systems, some manual work cannot be prevented; however, some operations could be further automated for example by using scripts. During the detailed analysis of shading caused by individual constructional parts of the machines, manual operations cannot be excluded. This concerns particularly the determination of boundaries between the cab and the movable or immovable parts of the machine within the total shading area for both scanner positions where the assessment and manual merging of the surfaces must be made for each position separately.

What appears to be very interesting is the temporal aspect of the data acquisition and the processing according to the mentioned procedure. The placement of the scanner in the defined position relative to the reference point of the seat and the subsequent measurement lasted approximately 20 min. The processing of data in the Faro Scene programme including the export took ca. 15 min. Time for data adaptation in the ArcGIS programme including the control analysis of the resulting raster and its transfer into the polygon could also be established in 15 min. An average CAD software user can import data from GIS to CAD, determine the total area of the vision and the vision obstruction, in addition to preparing the dimensions of the individual segments of the vision and the vision obstruction at a ground plan view distance of 12 m from the position of the driver’s eyes within 10 min. The boundaries between the cabin, the movable and immovable machine parts within the total shading area and their hatching could be made within another 5 min. The final manual process consists of filling the table with a numerical expression of the graphical output—ca. 5 min. The analysis can be considerably speeded up by using a drawing template with the prepared levels, the styles, the layouts for printing or exporting, and the vector objects of unchangeable parts of the output for the vision of the analysis according to the ISO standard requirements. Thus, the time of work in the CAD environment from the data import up to the printing takes approx. 20 min. The total actual time needed for the implementation of the TLS method from setting the scanner in the driver’s cabin up to the evaluation of the results in the graphic form takes approximately 70 min.

If we use the method of shadows cast by two point light sources, the time estimate of individual operations is as follows: installation of light sources into the defined position on the driver’s seat ca. 15 min. Recording of the shadow images cast from the left and right light sources onto the horizontal area with a square grid in the terrain plane takes about 35–50 min, depending on the complexity of the cast shadow images. Transcription of the obtained information into the field notes takes about 40–70 min, depending on the amount of data. The subsequent evaluation takes 70–100 min. Total net time required for carrying out the method of measuring the shadow images cast by two point light sources from the installation of light sources in the driver’s cabin up to the evaluation of the results in graphical form may range from 160 to 235 min. The implementation of the method requires collaboration of two persons. The times of both used methods were established based on the personal experience of the authors of the paper.

**Figure 7:**
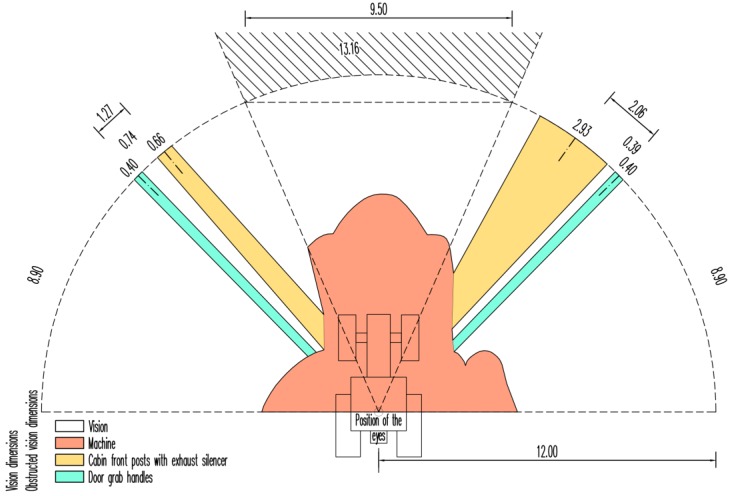
Semi-circular vision to the front of agricultural tractor driver determined by the method of measuring the cast of shade by two light point sources. Note: Dimensions are in *m*.

We compared our results obtained through the TLS method in the two machines with results obtained by using the method of shade by the two light point sources for the agricultural tractor (Figure 7, Table 3). The total length of the chords of section segments shaded by the front posts of the cabin and the exhaust silencer determined by the TLS method was lower by 19.22% and the difference in the cabin door grab handles was an even 30.00%. The area shaded by the front posts of the cabin and the exhaust silencer established by the TLS method was 7.57% lower and a similar situation was recorded in the shading of the remaining constructional elements (marked in the table as Machine) where the difference was 6.56%. The TLS method succeeded in capturing more details within the semi-circular driver’s field of vision, e.g., front screen wiper at the left front mudguard or the grab handles across the whole surface of the door. The facts showed, for example, in the increased surface area shaded by the door grab handles determined by TLS by 8.70% with the length of the chords of shading sections for this machine part being established lower by 30.00%. Complete information can be found in Table 4.

The situation was similar for the harvester driver’s field of vision. Comparing the two measuring methods, we found out the total length of sector segments shaded by the front posts of the cabin, the hydraulic crane and the hydraulic hoses, the screen wipers and the observation mirror was determined by the TLS method to be lower by 22.43%. The total surface area of shading was determined by the TLS method to be lower by 16.84%. As to the harvester driver’s field of vision, the greater differences of the measured values were recorded between the two methods. The fact could be explained by both the higher number and by the greater complexity of the constructional elements occurring within the harvester driver’s field of vision. The above facts indicate that the values of shading detected by the method of shade casting by light point sources are generally overestimated and more distorted for smaller constructional elements of the cabin.

Based on the above findings, we can formulate the advantages and the disadvantages of the two discussed methods determining the semi-circular field of the driver’s vision. The main advantages of the TLS method includes independence on the conditions of light and the speed of measurement in the field, which allows repeated measurements for various positions of machines and components. The high accuracy of this method facilitates the analysis of even smaller obstacles in the driver’s vision such as screen wipers, bundles of electrical cables, hydraulic hoses, etc. The method is less demanding in terms of the quality of the surface on which it is implemented. It can be used even on a surface, which is not smooth and exhibits irregularities, e.g., surfaces paved with gravel.

A disadvantage of the TLS method consists of the high purchasing cost of a scanner. This disadvantage can be remedied by ordering the measurements as a service from a specialized firm. Other disadvantages include the scanner sensitivity to soiled or scratched cabin screen glass and to the reduction of glass transparency due to heavy tinting. These glass treatments will manifest in a lower density of obtained points in the clouds, as in the case of a driver’s side vision shown in Figure 4. Problems may also be caused by the use of metal-coated foils that prevent the cabin from overheating in the summer. The processing of the measurement results requires software equipment, which allows for the described work operations.

One of the main advantages of using the light sources method in casting shaded images is its modesty in technical equipment. One only needs one or two light point sources and common measuring aids. No. specialized software is required for the evaluation.

The disadvantages include the time consumption factor and a necessity to take the measurements under the conditions of light, which allow for the identification of the deepest cast of shade. The measurement is done on a smooth surface (irregularities up to 25 mm per common metre) and with a marked square grid (1 m × 1 m). The method is less accurate and more susceptible to errors. Under the described specific conditions (conditions of light, sufficiently powerful point sources), it is sometimes difficult to precisely distinguish the boundaries of the deepest shade, namely in smaller obstacles.

## 6. Conclusions

The poor vision of a driver of an operating machine affects his labour performance during the shift and contributes to the impairment of conditions for safety and health protection during the work. The often-used method of determining the semi-circular driver’s field of vision is by measuring the shade cast by two light point sources. It is time consuming and conditions for the correct implementation of this method is stringent. The TLS method can significantly accelerate and partially automate the entire process of determining the driver’s vision while also improving its accuracy. Attention should be given to a scanner’s sensitivity to the parameters and to the mechanical damage of materials used for glass cabins.

Due to the high costs of instrumentation, the TLS method is suitable for specialized facilities such as machine testing stations or large manufacturers of mobile technology with their own developmental office. These can obtain quickly accurate information about the impact of an individual machine’s structural components on the driver’s view from the cabin by using the TLS method. Smaller companies are advised to use the method in the form of paid services.

According to our findings, the values measured by using the method of shadow images cast by two point light sources were overestimated. This is why we would consider it useful to verify these results by comparing the two methods to a statistically significant set of data.

During analyzing the field of driver’s vision, the standard ISO 5721-1 focuses on the chords of the shaded sectors of the semi-circular field of vision. Due to the nature of the industrial operations, which the drivers of operating machines have to perform under, it is in our opinion useful to also analyse the field of vision in an across-the-board way by using the above-described method. Nevertheless, the TLS method’s potential consists primarily in the spatial analysis of the driver’s vision combined with the method of analyzing the distribution of the driver’s attention to the individual’s vision of the field segments during the work shift. This subject will be discussed in follow-up papers by the authors.

## Figures and Tables

**Table 1:** Characteristics of the semi-circular vision to the front of agricultural tractor driver determined by the TLS method in units of area and length.

Agricultural Tractor			Area		Length at 12 m	
			m^2^	%	m	%
Vision			165.60	73.21	32.88	90.48
Obstructed vision	Machine		37.48	16.57	0.00	0.00
	Cabin front posts with exhaust silencer		16.02	7.08	2.90	7.98
	Door grab handles		7.10	3.14	0.56	1.54
Total			226.20	100.00	36.34	100.00

**Table 2:** Characteristics of the semi-circular vision to the front of harvester driver determined by the TLS method in units of area and length.

Harvester			Area		Length at 12 m	
			m^2^	%	m	%
Vision			161.34	71.33	31.01	83.56
Obstructed vision	Machine		44.51	19.68	2.38	6.41
	Cabin front posts		6.91	3.05	1.38	3.72
	Movable components		13.44	5.94	2.34	6.31
Total			226.20	100.00	37.11	100.00

**Table 3:** Characteristics of the semi-circular vision to the front of agricultural tractor driver determined by the method of measuring the cast of shade by two light point sources in units of area and length.

Agricultural Tractor			Area		Length at 12 m	
			m^2^	%	m	%
Vision			158.75	70.18	32.10	87.97
Obstructed ision	Machine		40.11	17.73	0.00	0.00
	Cabin front posts with exhaust silencer		21.16	9.35	3.59	9.84
	Door grab handles		6.18	2.73	0.80	2.19
Total			226.20	100.00	36.49	100.00

**Table 4:** Changes in the determination of area and length in the driver’s semi-circular field of vision established by using the TLS method as compared with the method of shadow images cast by point light sources.

Agricultural Tractor			Area	Length at 12 m
			%	%
Vision			4.31	2.43
Obstructed vision	Machine		−6.56	-
	Cabin front posts with exhaust silencer		−24.29	−19.22
	Door grab handles		14.89	−30.00
